# The importance of response generation protocols in chatbot-based patient education studies

**DOI:** 10.1590/1806-9282.20260715

**Published:** 2026-09-07

**Authors:** Muhammet Hüseyin Erkan

**Affiliations:** 1 Balikesir University, Department of Cardiovascular Surgery – Balıkesir, Turkey.

Dear Editor,

I read with great interest the article entitled “Artificial intelligence chatbots and idiopathic pulmonary fibrosis: are they ready to inform patients?”^1^ The study addresses a timely and valuable topic by evaluating widely used artificial intelligence (AI) chatbots as patient information tools for idiopathic pulmonary fibrosis. The authors’ assessment of chatbot responses in terms of readability, understandability, actionability, quality, and reliability provides an important contribution to the question of whether AI-based patient information tools are ready for clinical use.

The findings of the study show that AI chatbots may provide information that is generally concordant with guidelines; however, some limitations remain, particularly regarding readability, source transparency, and patient-oriented actionability. In this respect, the study represents a valuable starting point. Nevertheless, I believe that some methodological details, particularly regarding the protocol used to obtain chatbot responses, should be reported more explicitly, not because the findings are necessarily invalid, but because such details are important for reproducibility, interpretability, and comparison with future studies.

Although Şenel et al. reported several important methodological details, including the questions used, the AI chatbots evaluated, the query language, and the absence of human editing, some operational aspects of response generation remain unclear. These include account history, use of separate new chat sessions, exact prompt structure, and any procedure to minimize potential carry-over effects between queries. Clarifying these points would further improve reproducibility and comparability.

In studies evaluating AI chatbots, the accounts through which responses are generated represent an important methodological detail. Accounts previously used for different topics may affect the content of responses through prior conversation history or user-specific behavioral patterns. Therefore, in such methodological studies, it may be more appropriate to use newly created accounts that have not previously been associated with any AI model.

In addition, depending on the objective of the study, researchers should clearly define whether questions are submitted as originally formulated without additional instructions or whether standardized contextual prompts are used. If a zero-shot approach is preferred, the exact patient questions should be entered without additional explanations, role assignments, response-length instructions, or source-related prompts, and this approach should be explicitly reported.

Another important point is the use of a separate new chat session for each question. Consecutive questions asked within the same chat window may be influenced by previous question-answer pairs, which may limit the independent evaluation of each response. In previous AI chatbot evaluation studies, standardized response-generation procedures, including controlled query conditions, predefined patient-oriented questions, and clear reporting of model-access settings, have been used to improve reproducibility and comparability^2,3^. In addition, where repeated interactions with the same AI system or account are unavoidable, the inclusion of a predefined wash-out period between response-generation sessions may be considered as an additional precaution to minimize potential carry-over or contextual effects.

In conclusion, this study provides an important contribution to the evaluation of AI-based patient information tools. Future studies should explicitly report key response-generation details, including account status, prompt structure, use of separate chat sessions, and procedures to minimize carry-over effects. When these details are unclear, repeating selected queries under a predefined protocol may help assess the stability of the findings.

Thank you for the opportunity to provide feedback on this important work.

## Data Availability

The datasets generated and/or analyzed during the current study are available from the corresponding author upon reasonable request.
